# Supplementary material for: An Aegilops longissima NLR protein with integrated CC-BED module mediates resistance to wheat powdery mildew
Source: Nat Commun. 2024 Sep 27;15:8281. doi: 10.1038/s41467-024-52670-2 (PMC11436982; doi:10.1038/s41467-024-52670-2)
Supplement: Supplementary file 1 — Supplementary information [file 41467_2024_52670_MOESM1_ESM.pdf]

An *Aegilops longissima* NLR protein with integrated CC-BED module mediates resistance to wheat powdery mildew

Ma *et al.*

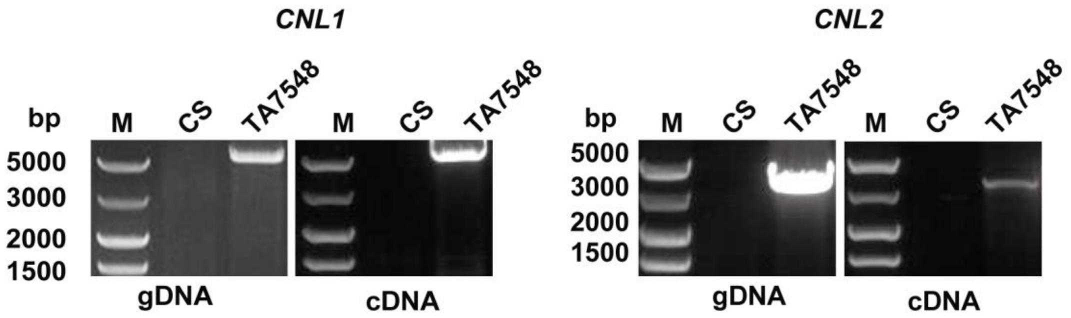

Supplementary Fig. 1. PCR amplification of full-length sequences of *CNL1* and *CNL2* in CS, TA7548 by primers *SALF-CNL1* and *SALF-CNL2*. M: 5 kb DNA Ladder Marker.

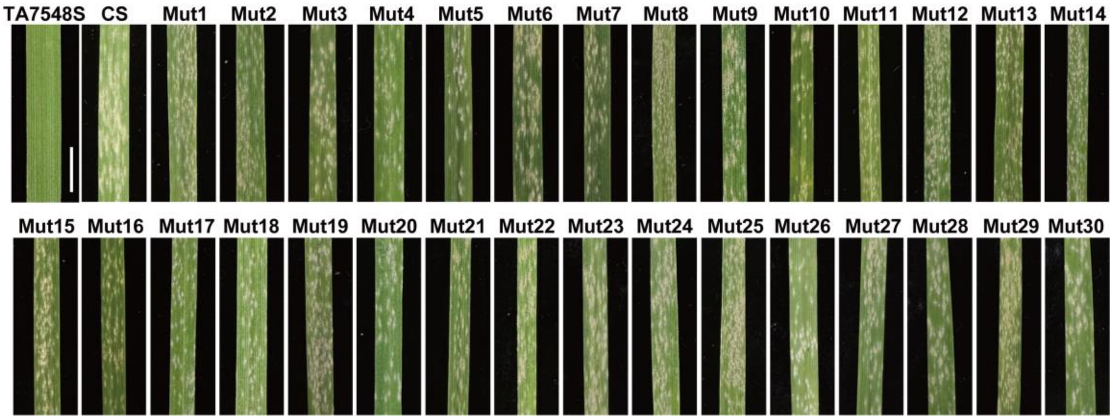

Supplementary Fig. 2. Resistant phenotype of leaf segments of susceptible mutants at 10 days post inoculation (dpi) with *Bgt* isolate E26. CS-*Ae. longissima* 6S^l#3[6A] substitution line TA7548S: resistance control, CS: susceptible control. Scale bar, 0.5 cm.

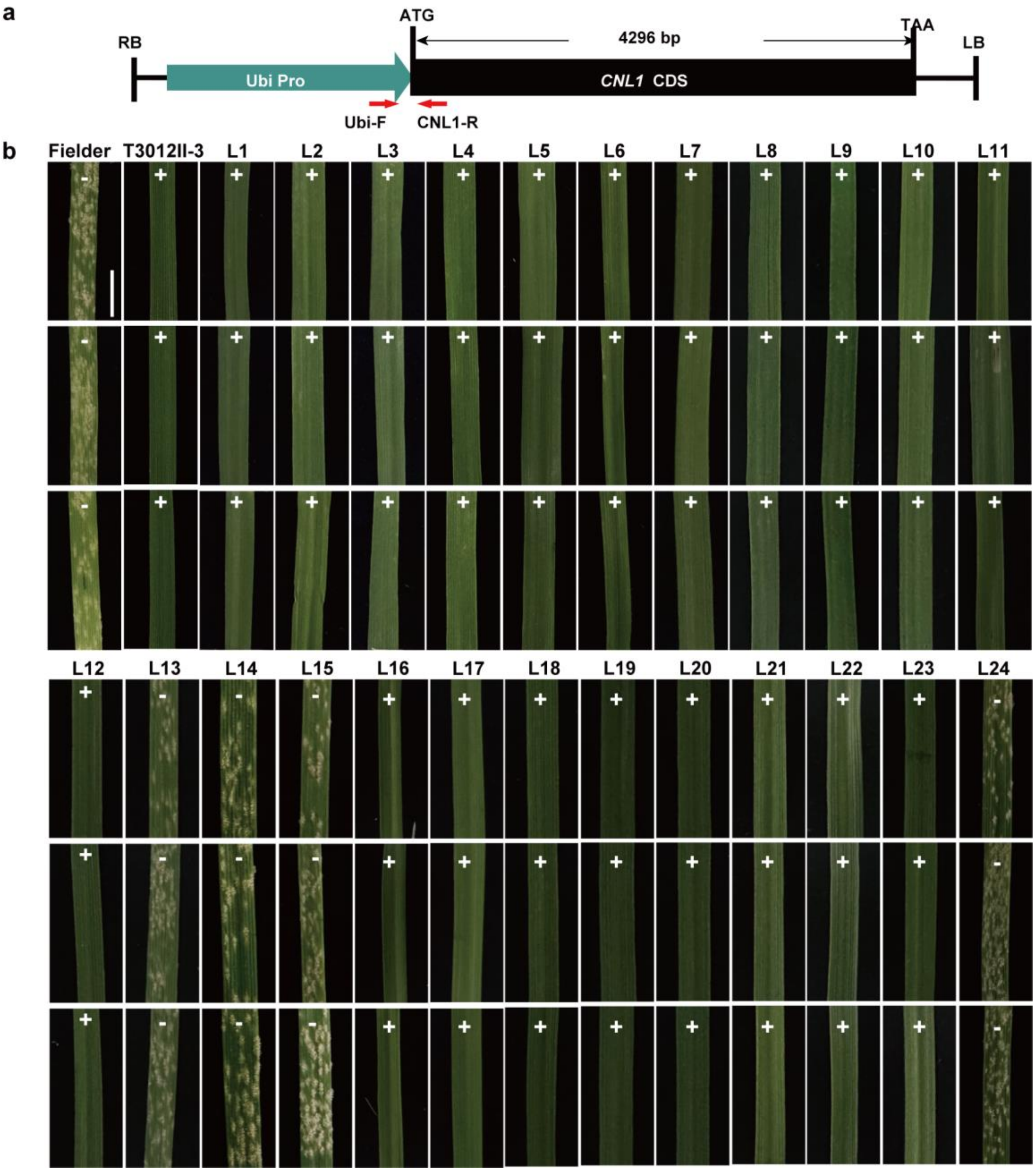

Supplementary Fig. 3. Genotype and phenotype of transgenic plants driven by the maize Ubi promoter. a Structure of pWMB110-*ProUbi*:*CNL1* vector used for *Agrobacterium*-mediated transformation. The red arrow indicates the positions of primer pairs *Ubi-F*/*CNL1-R* to detect *CNL1* in transgenic plants. **b** Resistance assay of transgenic T_1 lines 10 d post inoculation (dpi) with *Bgt* isolate E26, using receptor Fielder as a susceptible control and T3012II-3 as a resistance control. "+" indicates the presence of *CNL1*, while "−" indicates the absence of *CNL1*. The phenotype of three individuals from each T_1 line is shown. Scale bar, 0.5 cm.

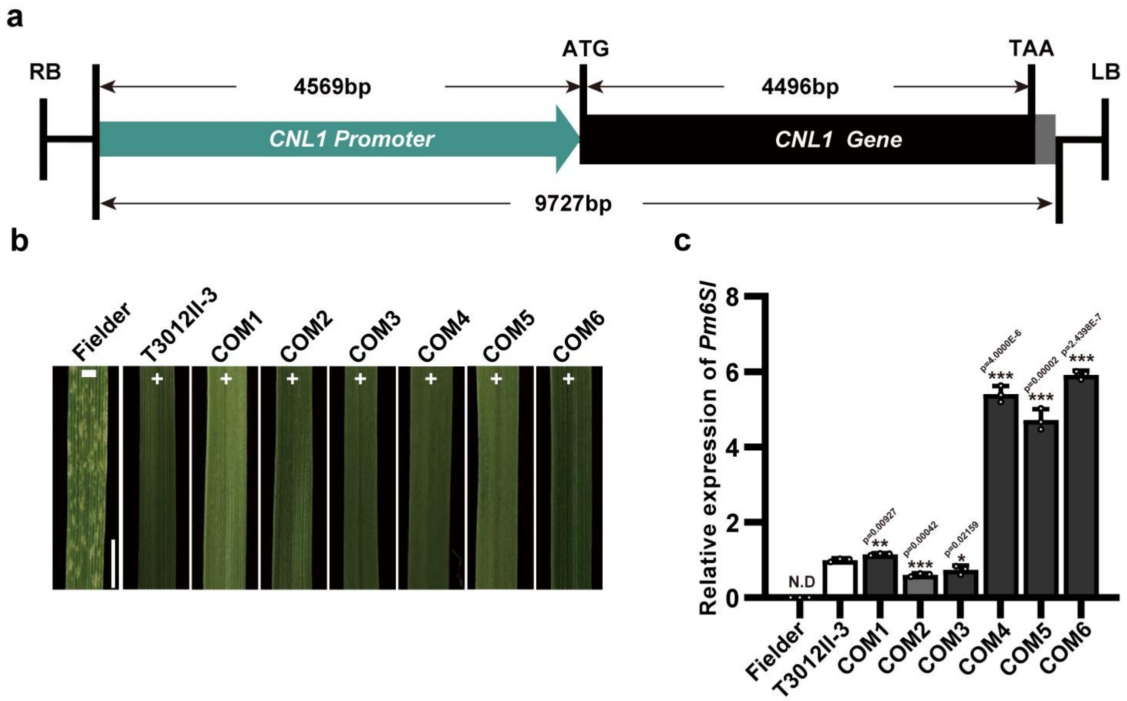

Supplementary Fig. 4. Genotype and phenotype of transgenic plants driven by the native promoter of *CNL1*. a Structure of *ProCNL1*:*CNL1* construct used for transgenic assay. The *ProCNL1:CNL1* construct contains the 4496 bp genomic DNA sequence of *CNL1*, 4,569 bp presumed promoter and 662 bp terminator. The *ProCNL1*:*CNL1* construct was transformed into *Agrobacterium tumefaciens* strain EHA105 and delivered into the wheat cultivar Fielder. **b** Resistance assay of transgenic T_0 plants 10 d post inoculation (dpi) with *Bgt* isolate E26, using receptor Fielder as susceptible control and T3012II-3 as resistance control. A total of six transgenic T_0 seedlings (COM1-6) were obtained. From each T_0 seedling, a completely expanded new leaf was selected, one half for expression level analysis and the other half for powdery mildew resistance assessment. Marker *pm6Sl-1* was used to detect *CNL1* in the transgenic plants. "+" indicates the presence of *CNL1*, while "−" indicates the absence of *CNL1*. Scale bar, 0.5 cm. **c** Transcript levels of *CNL1* in the leaves of 6 T_0 transgenic seedlings (COM1-6) and the *Pm6Sl* introgression line T3012II-3. Expression levels were examined by qRT–PCR with *TaACTIN* as endogenous control and calculated using the comparative CT method. The data are presented as the means ± SDs from three technical replicates. N.D: not detected. Asterisks (*, **, ***) represent significant differences at $p < 0.05$, $p < 0.01$ and $p < 0.001$ levels, respectively (two-tailed Student’s *t* test). Source data is provided as a Source Data file.

a

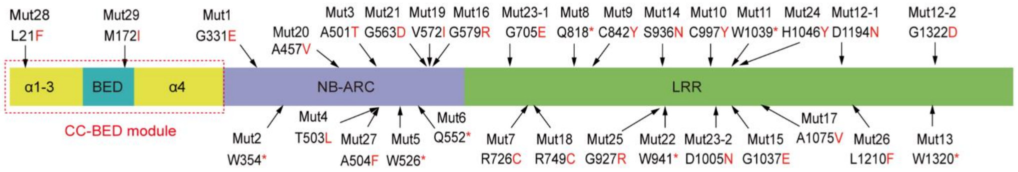

b

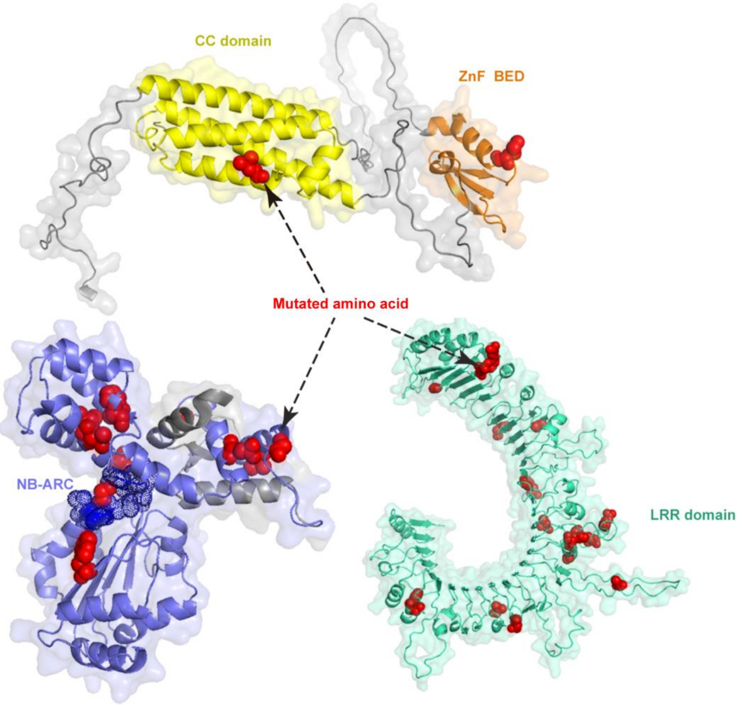

Supplementary Fig. 5. The amino acid (AA) changes at mutation sites in Pm6Sl protein. **a** The amino acid (AA) changes at mutation sites in Pm6Sl domains. **b** The amino acid (AA) changes at mutation sites in 3D structure of Pm6Sl protein, mutated AA were indicated by red spheres.

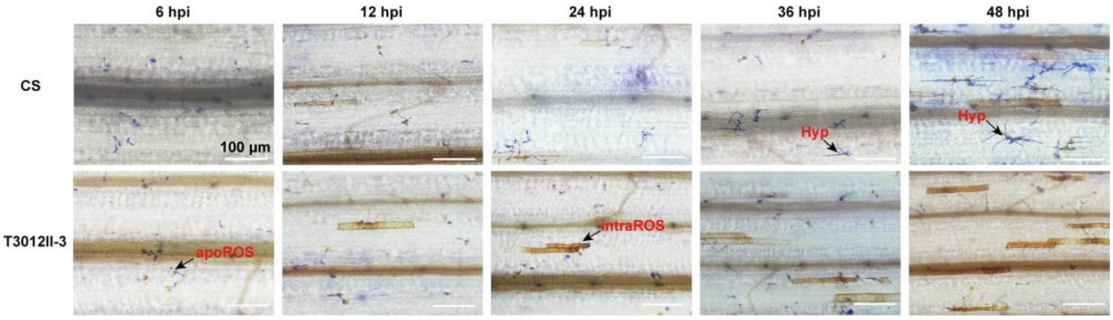

Supplementary Fig. 6. DAB-Coomassie blue-stained leaf sections inoculated with *Bgt* isolate E26. Brown staining indicates intracellular reactive oxygen species (IntraROS), while blue staining shows *Bgt* fungus. apoROS: apoplastic ROS. Hyp: hyphae. Scale bar, 100 µm.

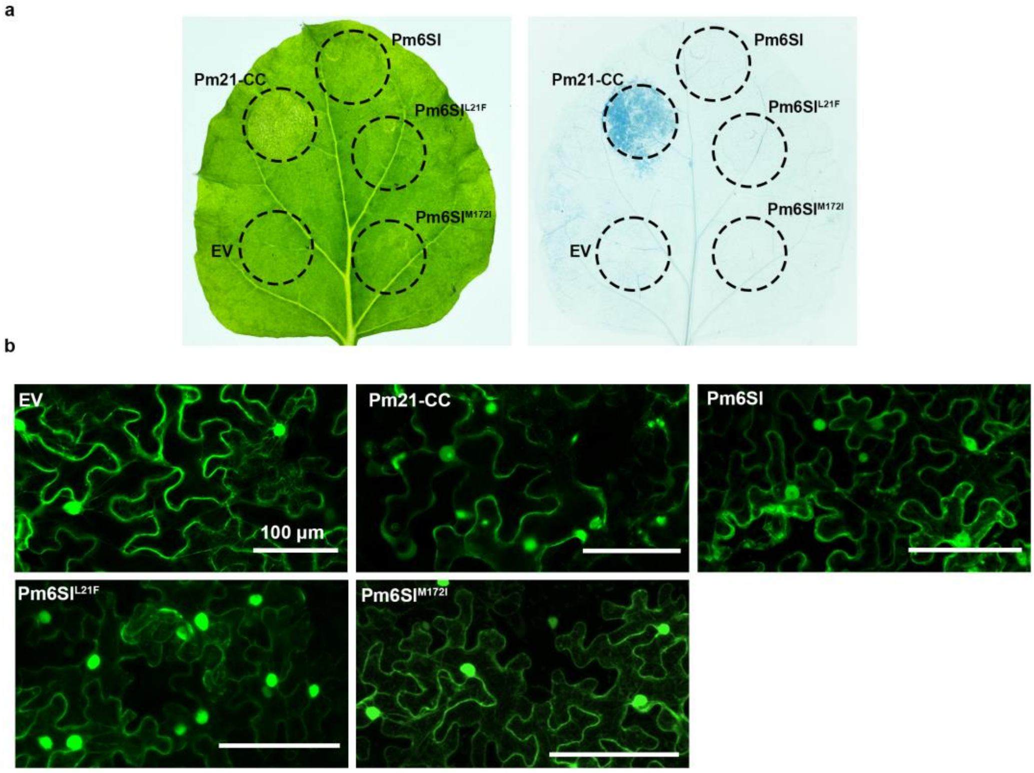

Supplementary Fig. 7. Lethal ability analysis and expression of GFP fusion protein of Pm6Sl protein and mutations in *N. benthamiana* leaves. a Transient expression, trypan blue (TPN) staining of Pm6Sl full length protein and two mutations identified in the Pm6Sl CC-BED domain through EMS mutagenesis in *N. benthamiana* cells. **b** The expression of fusion protein of full-length Pm6Sl and two mutations with GFP in *N. benthamiana* cells. The outcomes of fluorescence signal detection confirmed the successful expression of these fusion proteins.

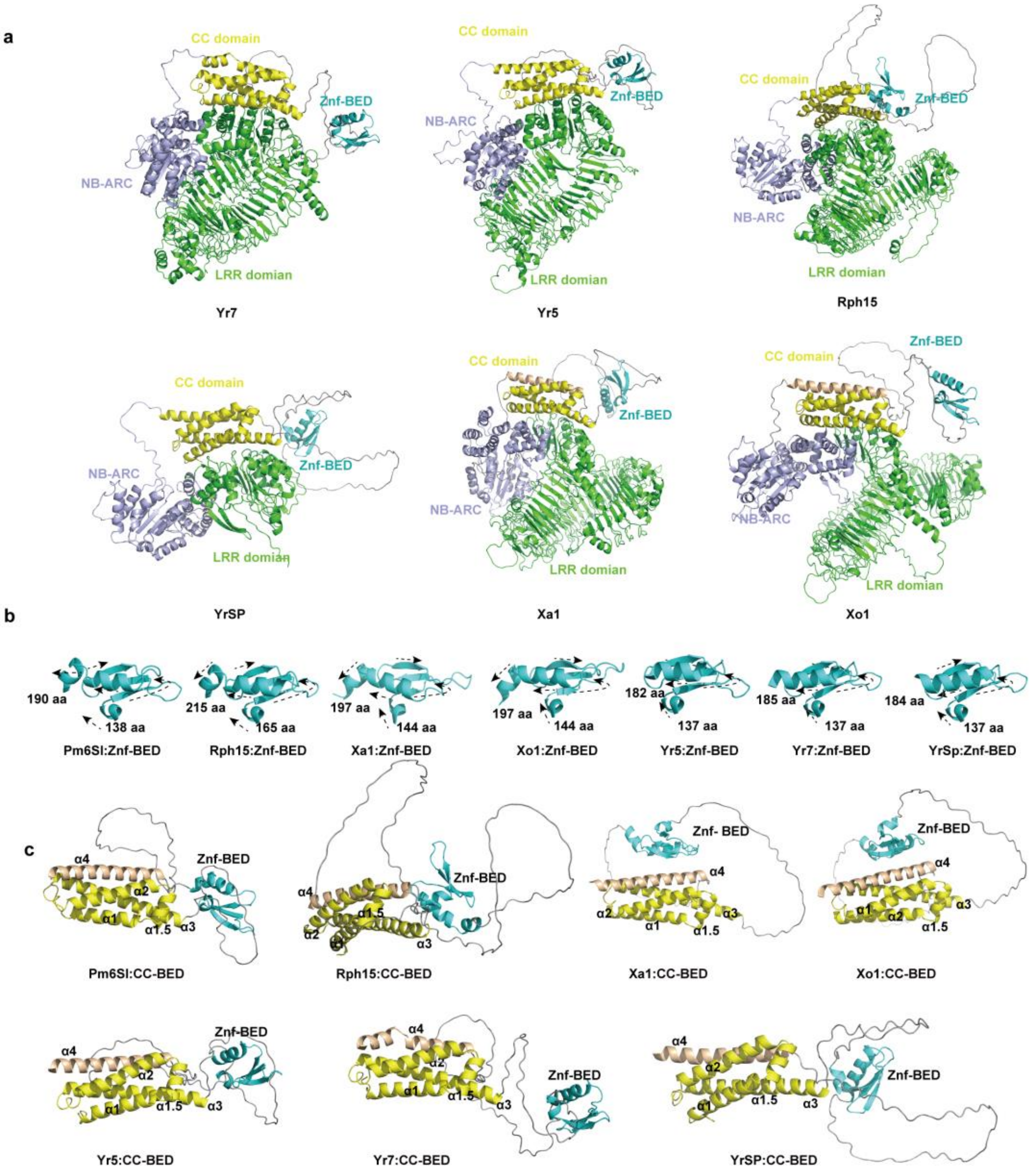

Supplementary Fig. 8. 3D Structure of six crop BED-NLRs and their Znf-BED domains, CC-BED modules predicted using AlphaFold2. Full length (**a**), Znf-BED domains (**b**) and CC-BED modules (**c**) of six BED-NLRs.

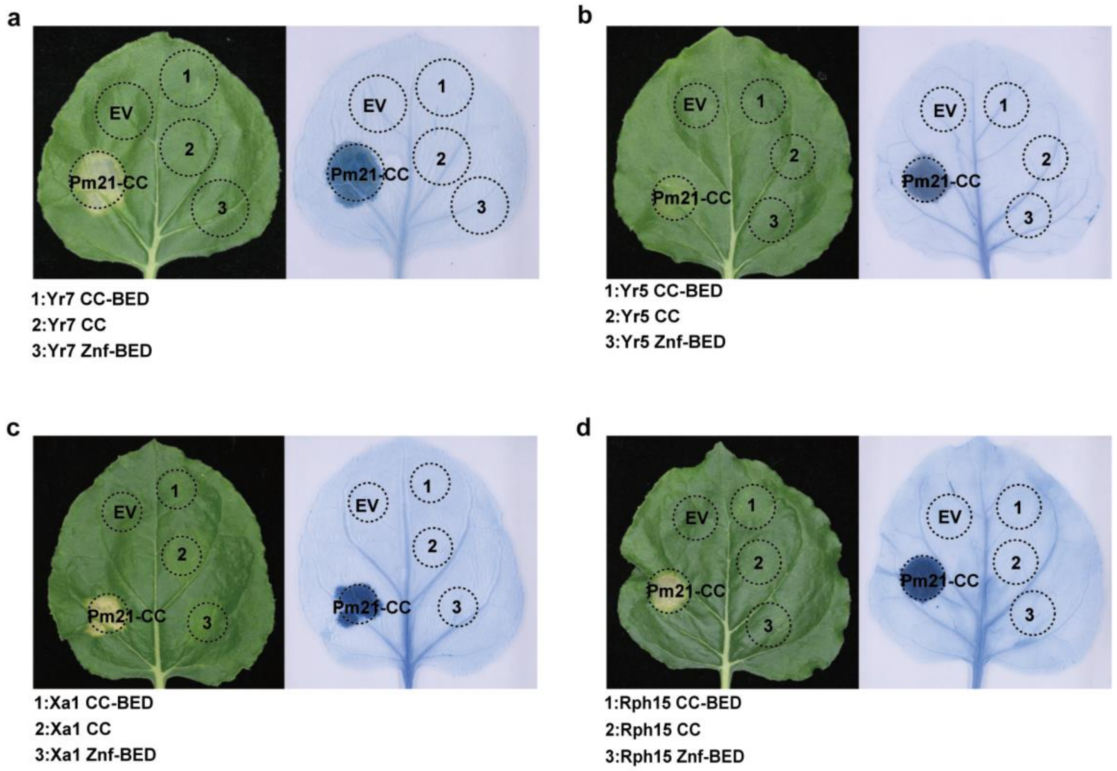

Supplementary Fig. 9. Transient expression and trypan blue staining of truncated CC-BED modules of Yr7/Yr5/Xa1/Rph15 in *N. benthamiana* cells. a Transient expression and trypan blue (TPN) staining of the CC-BED, CC and Znf-BED domains of Yr7 in *N. benthamiana* cells. **b-d** Transient expression and TPN staining of the truncated CC-BED modules from Yr5 (b), Xa1 (c) and Rph15 (d), respectively. Empty vector (EV), negative control; Pm21-CC, positive control. The results revealed that none of the truncated CC-BED modules of Yr7/Yr5/Xa1/Rph15 induced cell death.

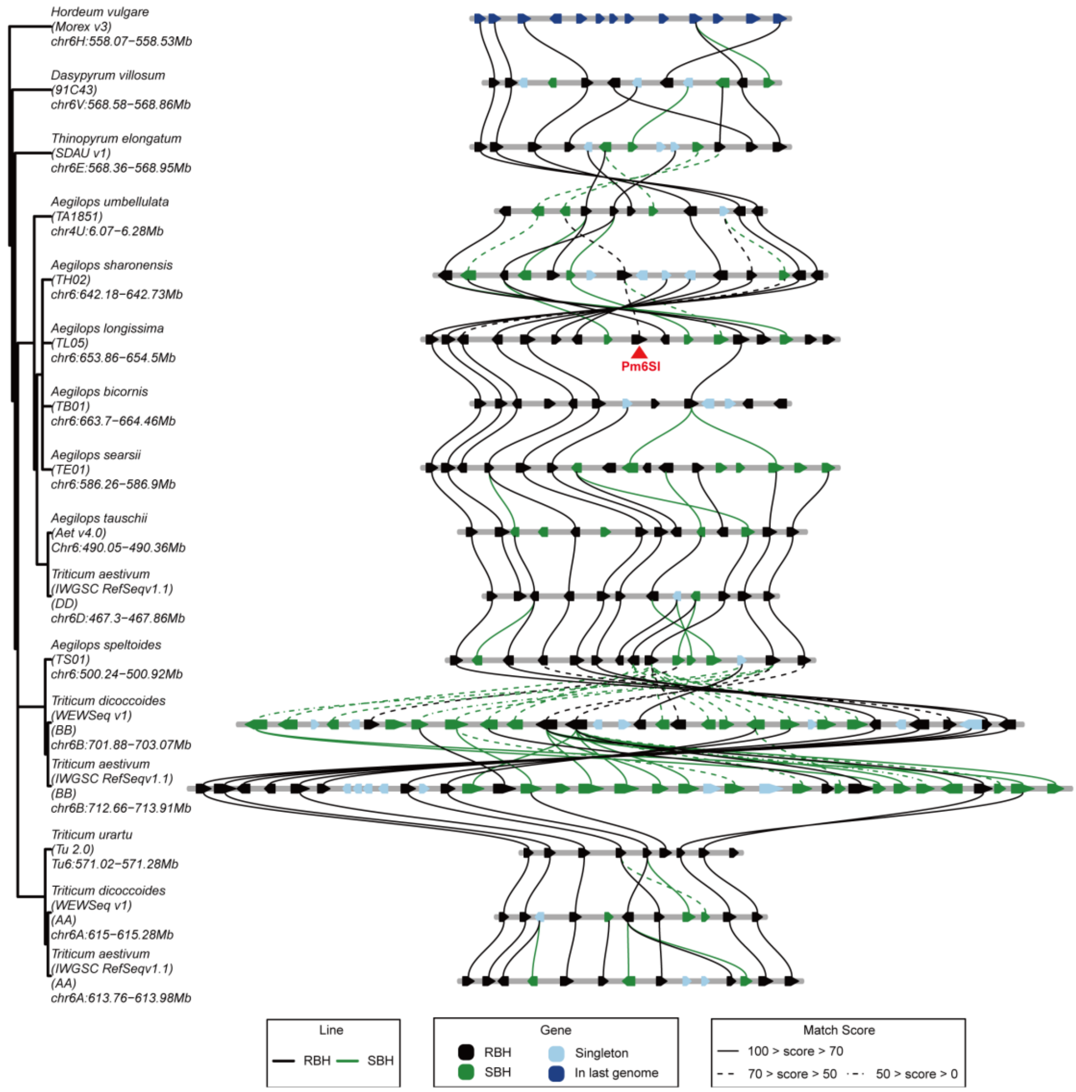

Supplementary Fig. 10. Collinearity of *Pm6Sl* genomic regions in homoeologous group 6 chromosomes of wheat related species. RBH (reciprocal best hit), SBH (single-side best hit) and singleton indicated three different types of homologous relationships. RBH: gene pairs belonging to the Reciprocal Best Hits; SBH: gene pairs belonging to the Single-side Best Hits, where RBH is not found but the best matching gene is found; Singleton: the genes with no homologous genes. The left tree is obtained from a pre-computed relationship derived from Triticeae-GeneTribe.

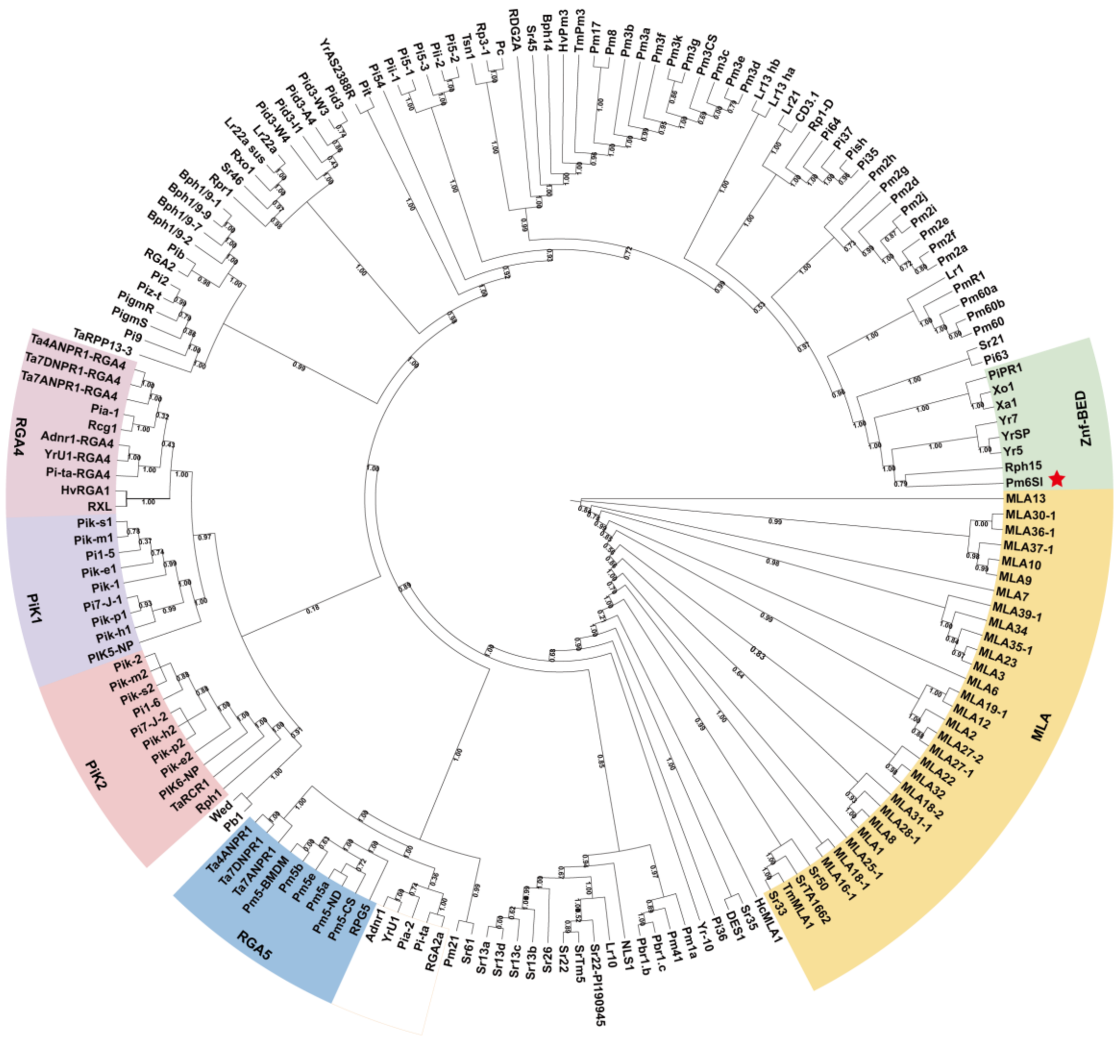

Supplementary Fig. 11. Phylogenetic tree of Pm6Sl and other NLR Proteins in Gramineae. Phylogenetic analysis of Pm6Sl with 180 other proteins from Gramineae family used to analyze the CNL network by Contreras *et al.*[1]

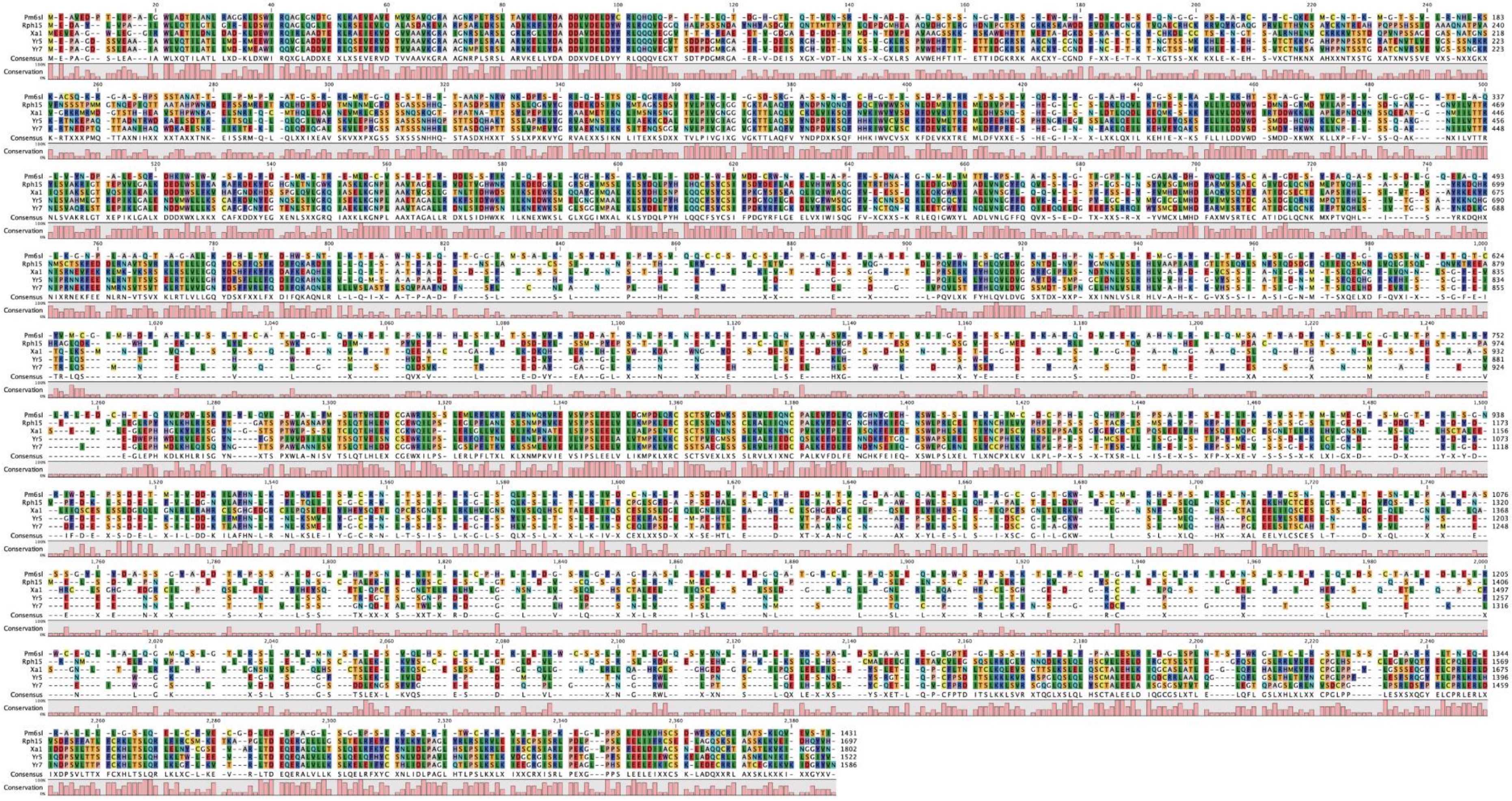

Supplementary Fig. 12. Comparison of amino acid sequences of Pm6Sl with Rph15, Xa1, Yr5, Yr7. The same amino acids are coded in the same color**.**

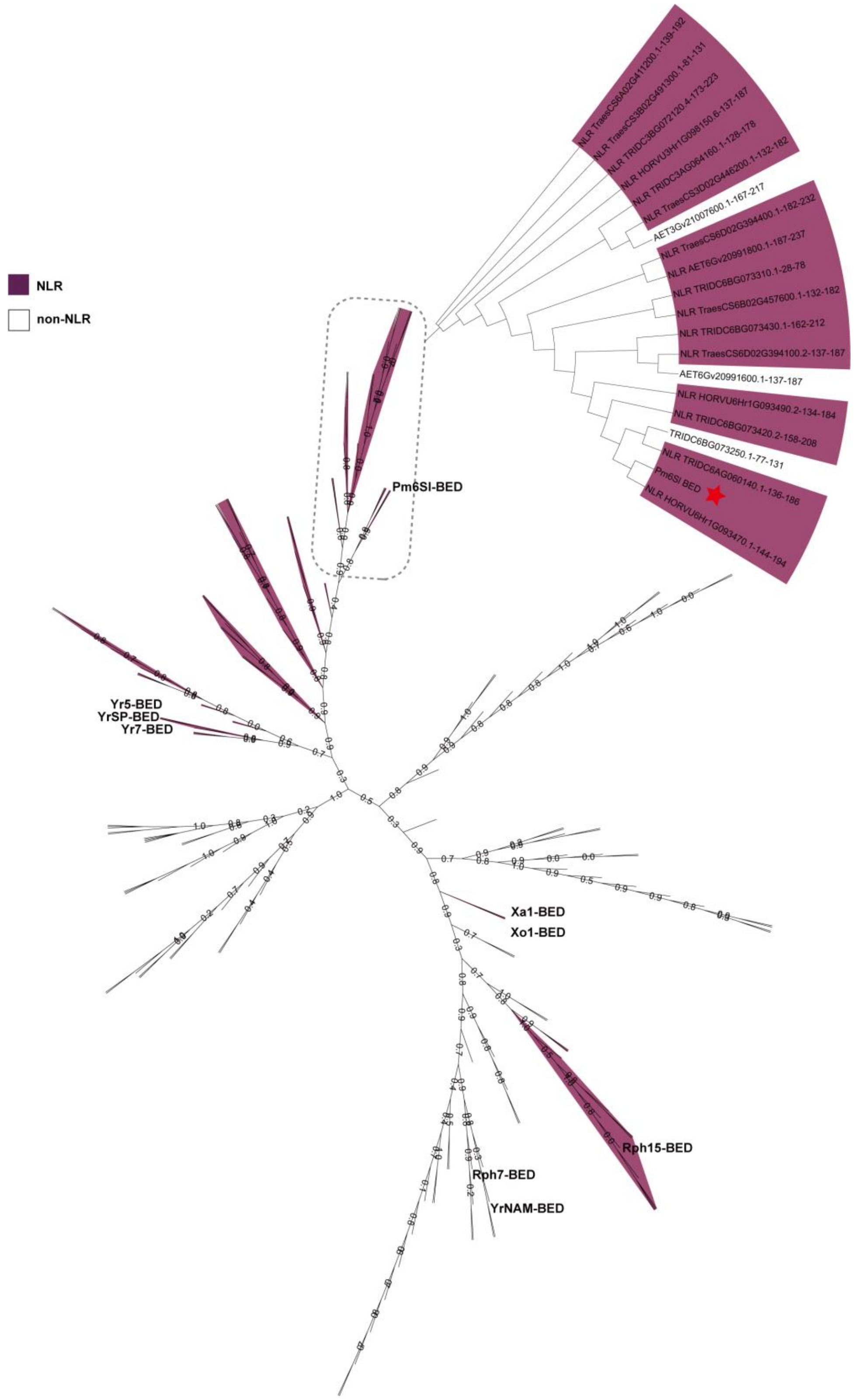

Supplementary Fig. 13. Phylogenetic tree of Znf-BED domains. Phylogenetic analysis of Znf-BED domains of Pm6Sl and six other crop BED-NLRs, along with other 154 non-NLR and NLR proteins containing Znf-BED domains in Gramineae family reported by Marchal *et al*.[2].

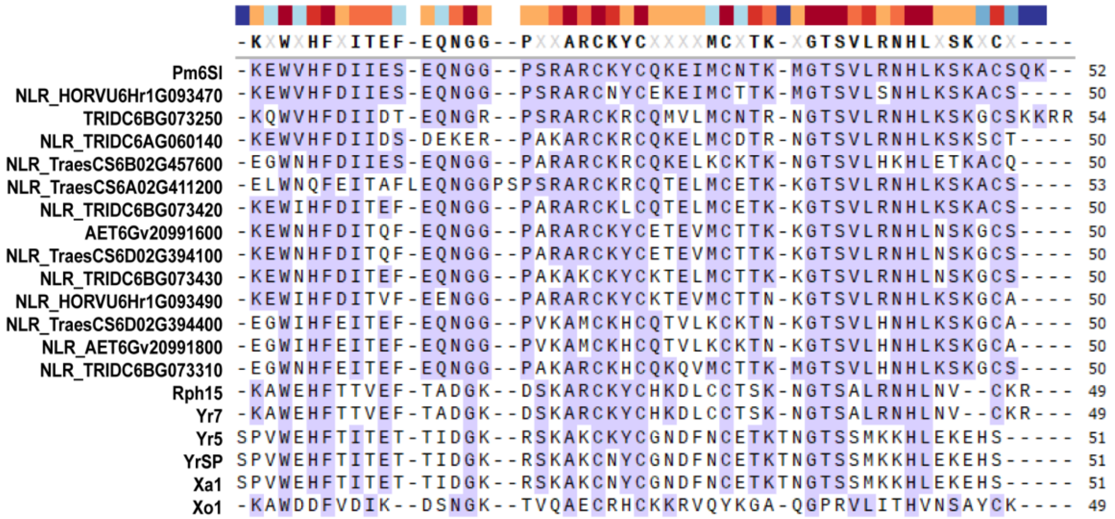

Supplementary Fig. 14. Alignments of Znf-BED domains from Pm6Sl with proteins annotated in homoeologous group 6 chromosomes of wheat-related species, and six cloned BED-NLR proteins. Residues that match those in Pm6Sl are highlighted by bright purple shading.

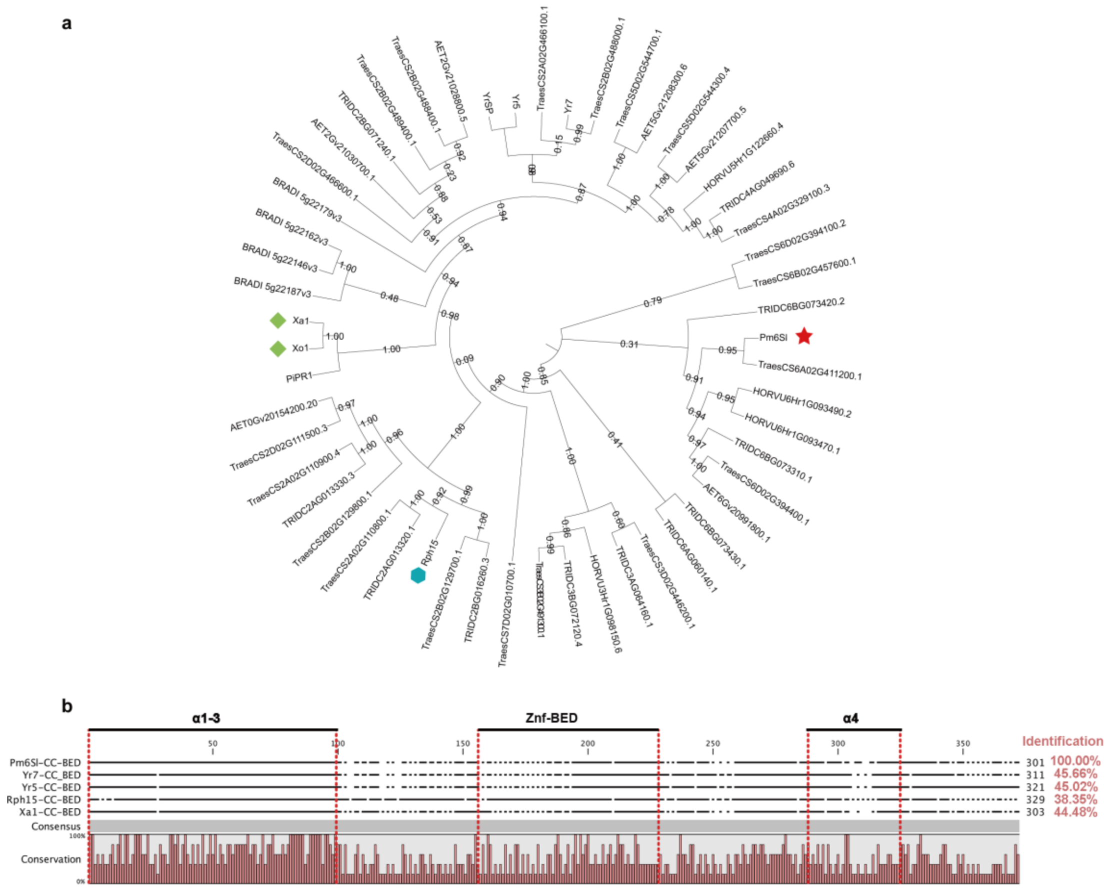

Supplementary Fig. 15. Phylogenetic tree of Pm6Sl CC-BED module with those of other BED-NLRs. a Phylogenetic analysis of Pm6Sl CC-BED module and six other crop BED-NLRs, along with 46 NLR proteins containing Znf-BED domains used in Supplementary Fig. 13. **b** Sequence conserved site and similarity analysis of Pm6Sl CC-BED module with Yr7/Yr5/Rph15/Xa1.

Supplementary references

1. Contreras, M. P., Ludke, D., Pai, H., Toghani, A. A. & Kamoun, S. NLR receptors in plant immunity: making sense of the alphabet soup. *EMBO Rep.* **24**, e57495 (2023).
2. Marchal, C., Haberer, G., Spannagl, M. & Uauy, C. Comparative genomics and functional studies of wheat BED-NLR loci. *Genes (Basel)* **11**, 1460 (2020).
