# Supplementary material for: An Aegilops longissima NLR protein with integrated CC-BED module mediates resistance to wheat powdery mildew
Source: Nat Commun. 2024 Sep 27;15:8281. doi: 10.1038/s41467-024-52670-2 (PMC11436982; doi:10.1038/s41467-024-52670-2)
Supplement: Supplementary file 3 — Description of Additional Supplementary Files [file 41467_2024_52670_MOESM3_ESM.pdf]

Description of Additional Supplementary Files

File Name: Supplementary Data 1
Description: Genotypes and resistance phenotypes of CS-*Aegilops longissima* $6S^{l}$#3 recombinants.

File Name: Supplementary Data 2
Description: List of primers used in the study.

File Name: Supplementary Data 3
Description: Seven genes annotation in the 210 kb interval based on *Ae. longissima* cv. TL05 reference genome sequence.

File Name: Supplementary Data 4
Description: Sequences of *CNL1*.

File Name: Supplementary Data 5
Description: Sequences of *CNL2*.

File Name: Supplementary Data 6
Description: List of mutants.

File Name: Supplementary Data 7
Description: Pm6Sl protein blastp in the NCBI non-redundant protein sequence database.

File Name: Supplementary Data 8
Description: List of plant materials used in the study.

File Name: Supplementary Data 9
Description: Responses of TA7548 and T3012II-3 to *Bgt* isolates.
